# Supplementary material for: The interactome of intact mitochondria by cross-linking mass spectrometry provides evidence for coexisting respiratory supercomplexes
Source: Mol Cell Proteomics. 2017 Dec 8;17(2):216–32. doi: 10.1074/mcp.RA117.000470 (PMC5795388; doi:10.1074/mcp.RA117.000470)
Supplement: Supplemental Data [file supp_RA117.000470_133922_0_supp_23978_fzffwf.zip › spectra_annotation/mito_spectra_annotation/279-1-1-1-8-1.pdf]

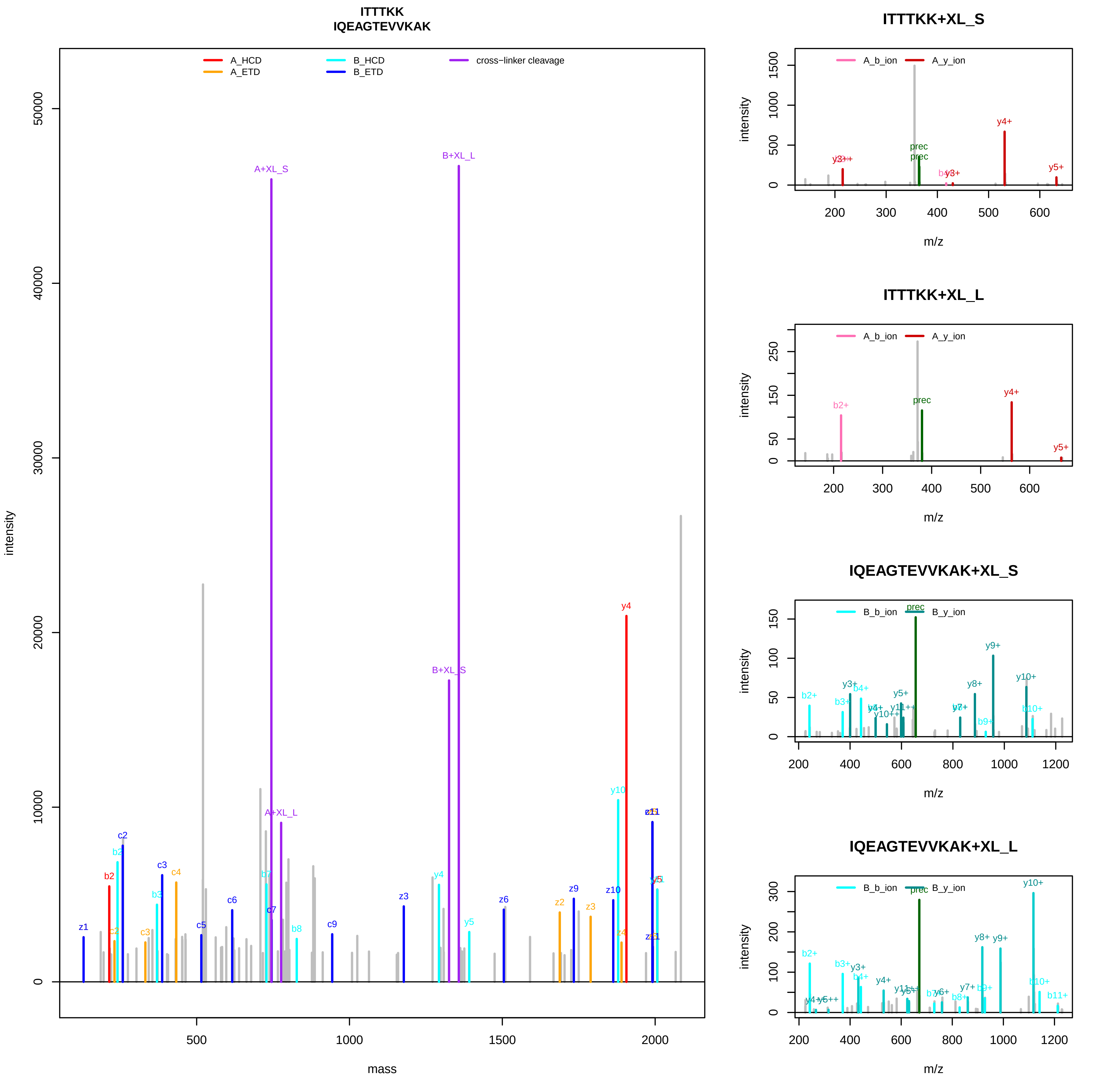
ITTTKK
IQEAGTEVVKAK
A_HCD
A_ETD
B_HCD
B_ETD
cross−linker cleavage
A+XL_S
B+XL_L
B+XL_S
A+XL_L
y4
y10
z11
z10
z9
z6
z3
z2
z3
c9
c7
c6
c5
c4
c3
c2
b2
b3
b7
b8
y5
z1
intensity
mass
0
10000
20000
30000
40000
50000
500
1000
1500
2000
ITTTKK+XL_S
A_b_ion
A_y_ion
y4+
y5+
y3+
prec
m/z
ITTTKK+XL_L
b2+
prec
IQEAGTEVVKAK+XL_S
B_b_ion
B_y_ion
y9+
y10+
y8+
y3+
b4+
y5+
b2+
b3+
b9+
b10+
IQEAGTEVVKAK+XL_L
y10+
y8+
y9+
b2+
b3+
y3+
b4+
y4+
y7+
b11+
